# Using search engine big data for predicting new HIV diagnoses

**DOI:** 10.1371/journal.pone.0199527

**Published:** 2018-07-12

**Authors:** Sean D. Young, Qingpeng Zhang

**Affiliations:** 1 University of California Institute for Prediction Technology, Department of Family Medicine, University of California Los Angeles, Los Angeles, California, United States of America; 2 Department of Systems Engineering and Engineering Management, City University of Hong Kong, Kowloon, Hong Kong; Hokkaido University Graduate School of Medicine, JAPAN

## Abstract

**Background:**

A large and growing body of “big data” is generated by internet search engines, such as Google. Because people often search for information about public health and medical issues, researchers may be able to use search engine data to monitor and predict public health problems, such as HIV. We sought to assess the feasibility of using Google search data to analyze and predict new HIV diagnoses cases in the United States.

**Methods and findings:**

From 2007 to 2014, we collected search volume data on HIV-related Google search keywords across the United States. State-level new HIV diagnoses data were collected from the Centers for Disease Control and Prevention (CDC) and AIDSVu.org. We developed a negative binomial model to predict HIV cases using a subset of significant predictor keywords identified by LASSO. The Google search data were combined with state-level HIV case reports provided by the CDC. We use historical data to train the model and predict new HIV diagnoses from 2011 to 2014, with an average R^2^ value of 0.99 between predicted versus actual cases, and average root-mean-square error (RMSE) of 108.75.

**Conclusions:**

Results indicate that Google Trends is a feasible tool to predict new cases of HIV at the state level. We discuss the implications of integrating visualization maps and tools based on these models into public health and HIV monitoring and surveillance.

## Introduction

More than 1.1 million people in the United States are living with HIV, with approximately 1 in 7 of them unaware of their infection [1]. Innovative methods are needed to increase HIV testing to prevent the spread of HIV [2].

Some of the most common current methods for HIV monitoring and surveillance include behavioral risk surveys, interviews, and laboratory testing reports [3]. One limitation of these existing methods is that there is typically a lag time in reporting before data are publicly released [4]. Another limitation is that data collection and aggregation methods require extensive time and financial resources [5].

New technologies might be incorporated into HIV monitoring methods to augment and address the limitations of existing tools [5,6]. For example, social media (e.g., Twitter) data have been used to predict seasonal illness [7–9] and HIV [5]. Search engine data have also shown to be potentially useful in public health research, as people are frequently seeking medical information on the internet and these aggregate search data are publicly available [10]. For example, researchers have explored different methods of using Google searches to predict influenza [11–14], monitor infectious diseases [15–17], and predict opioid-related emergency department visits for heroin [18]. Because historical state-level HIV data are publicly available, it may be possible to conduct a longitudinal analysis using these “big data” to determine whether search engine data can be used to predict new HIV case diagnoses [19].

This study sought to determine whether Google Trends [20] search data could be used to predict HIV. Public health data modeling of this type, if successful, could help to provide health agencies with information that typically becomes available more than one year later, allowing them to allocate resources earlier and more efficiently to needed areas.

## Methods

This study was approved the UCLA human subjects review board (reference number: 16–001275). We collected Google Trends relative search volume data for 22 HIV risk-related (both sexual and drug-related) keywords (e.g., sex, alcohol, HIV, cocaine). More information about the keywords is described online from a previous study [5]. Keywords were used for Google searches and were stemmed and could be used in a variety of formats within search behavior. For example, “HIV” could be used in searches as “where to get an HIV test” or “what are the symptoms of HIV?”

State-level HIV case diagnoses for each state from 2008 through 2014 were downloaded from AidsVu.org [21]. AidsVu produces an interactive map that displays HIV prevalence and case diagnoses by county in the United States. The United States Centers for Prevention and Disease Control (CDC) and national HIV surveillance database supply HIV data to AidsVu based on zip code [4]. Additionally we included the GINI index, an indicator of wealth inequality, as an additional covariate.

To model the number of HIV cases, we used a negative binomial generalized linear model (nbGLM), which is a widely adopted statistical model for count data [22]. Because new HIV case diagnoses are influenced by the preceding year’s cases, we also included a first-order autoregressive term in the model to capture this temporal pattern. Formula (1) presents the formation of the nbGLM model:

$$ln({y(t)}_{s})=\mu_{0}+\sum_{n=1}^{22}\mu_{n}ln({G(t)}_{n,s})+{\mu_{23}GINI(t)}_{s}+{\mu_{24}ln(y(t-1)}_{s})+\sum_{p=1}^{50}\alpha_{p}\;S_{p,s}+\varepsilon_{t},$$ (1)

where *y*(*t*)_*s*_ and *y*(*t* − 1)_*s*_ represent the new HIV diagnoses cases of state *s* in year *t* and *t* − 1, respectively; *G*(*t*)_*n,s*_ represents the data of the *n_th_* Google search term of state *s* in year *t*; *GINI*(*t*)_*s*_ represents the GINI coefficient of state *s* in year *t*; *S_p,s_* is the dummy variable, representing one state; *ε_t_* represents the white noise.

To identify the subset of Google keywords with the best predictive power, we adopted the Least Absolute Shrinkage and Selection Operator (LASSO) method to eliminate keywords that were not significant predictors [23]. The penalty coefficient (lambda) that leads to the best fitting result was chosen for each prediction. The LASSO based keyword selection was updated for each state for every year. To predict the count of HIV cases for a specific state in next year *t*, we used all historical data from 2008 to t-1 for model training. Because we have an autoregressive term in the model, we started from the prediction of HIV cases in 2011 using training data of 2008–2010. We then used the model to predict new HIV cases from 2011 to 2014.

### Data analysis

To evaluate the accuracy of the proposed model, we adopted the commonly used root-mean-square error (RMSE), which measures the deviations of predicted values from actual observed values. The smaller the RMSE is, the more accurate the model is. Because the use behaviors of Google Search change over time, the relevant keywords could be different every year. Therefore, our model was designed to be updated every year.

## Results

The RMSE, R, R^2^, and average RMSE for best fitting nbGLM-LASSO model for validation years 2011 through 2014 are shown in Table 1. The best fitting model had an R^2^ greater than 0.99 for all four validation years. The average coefficient of each variable is presented in Table 2, which also presents the proportion of states in which each variable is determined to be significant by the LASSO method. As expected, the autoregressive term is significant for all years, as the aggregated yearly count of HIV cases for a state is unlikely to change dramatically. We trained the model using an adaptive time window, rather than a fixed time window, because only one search term was significant for all years, indicating that the correlation between Google search data and actual new HIV cases is dynamic.

**Table 1:** Results of using Google Trends-based model to predict new HIV case diagnoses from 2011–2014.

	2011	2012	2013	2014	RMSE(Avg.)
**RMSE**	109.85	109.674	62.394	193.1	118.75
**R**	0.997	0.998	0.999	0.995	
**R**^**2**^	0.995	0.997	0.997	0.991	

RMSE = Root-mean-square error.

**Table 2:** Average coefficient of each variable (excluding control variables) and the proportion of states in which the variable is significant.

Variable	Prediction for 2011	Prediction for 2012	Prediction for 2013	Prediction for 2014	Significant proportion
**x1**	0	0	0	0	0%
**x2**	0	0	0	0	0%
**x3**	-0.05	0	0	0	25%
**x4**	0	0	0	0	0%
**x5**	-0.04	-0.03	0	0	50%
**x6**	0	0	0	0	0%
**x7**	0	0	0	0	0%
**x8**	0	0	0	0	0%
**x9**	0.02	0.01	0	0	50%
**x10**	0.02	0.01	0	0	50%
**x11**	0	0	0	0	0%
**x12**	0	0	0	0	0%
**x13**	0	0	0	0	0%
**x14**	0	0	0	0	0%
**x15**	0	0	0.01	0	25%
**x16**	0	0	0.01	0.01	50%
**x17**	-0.17	-0.11	-0.07	-0.05	100%
**x18**	0	0	0	0	0%
**x19**	0	0	0	0	0%
**x20**	-0.03	0	0	0	25%
**x21**	0	0	0	0	0%
**x22**	0.01	0	0	0	25%
**gini**	0.45	0.56	0	0.36	75%
**AR1**	0.95	0.97	0.98	0.98	100%

**Fig 1:**
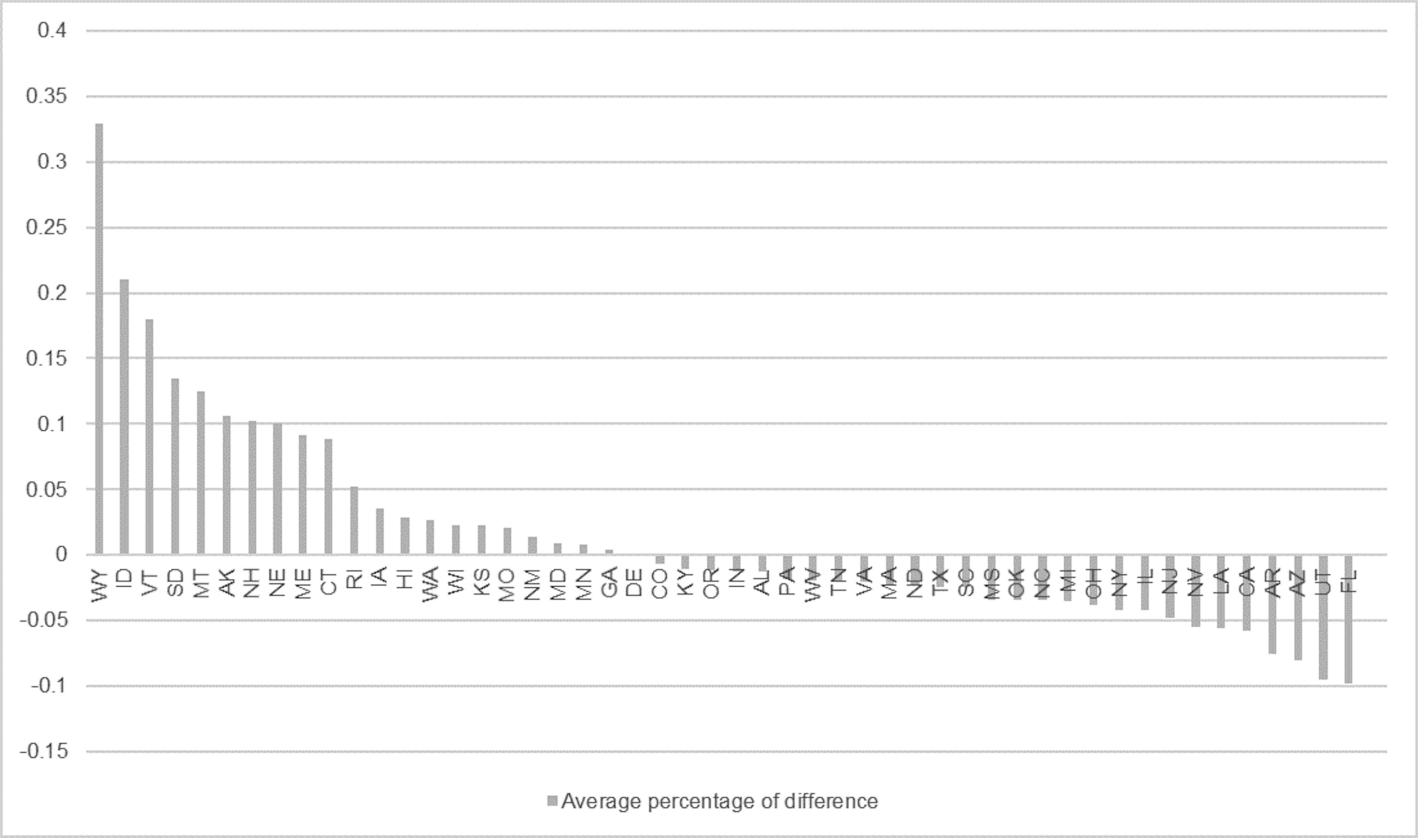
The average percentage of difference (forecast error) for each state (2011 to 2014).

Fig 1 presents the average percentage of difference, or forecast error (predicted value minus observed value), for all states during the periods where we predicted new case diagnoses (2011 to 2014). The model achieved a very small difference (<10%) for most states, except for WY, ID, VT, MT, AK, and NH, where the model overestimated the incidence, likely due to the lower population and lower number of HIV cases (ranging from only a few dozen to a hundred per year) within these states, making the prediction task more difficult and with higher percentage of difference. For more details about the prediction results, please refer to S1 Table.

## Discussion

This study suggests that internet search query data can be used to predict new HIV diagnoses cases across the United States. We decided to use Google Trends data as an indicator for a predictive model based on the hypothesis that people at risk for HIV would be likely to search the internet for HIV-related topics, including risk behaviors, prevention, and testing information. Internet search engine data have quickly become a rich source of “big data,” allowing researchers to use data on search behavior to help predict HIV and other illnesses.

The results of this study are important because they support including models and visualizations of data based on freely available internet social data that are not readily captured by current methods. Although an increasing number of studies are incorporating social data into public health research, few studies have focused on how to integrate these approaches to address the issues related to HIV [24]. This study furthers the field of study on use of social data in HIV research by suggesting that Google search data can be used to predict new HIV case diagnoses across the United States.

**Fig 2:**
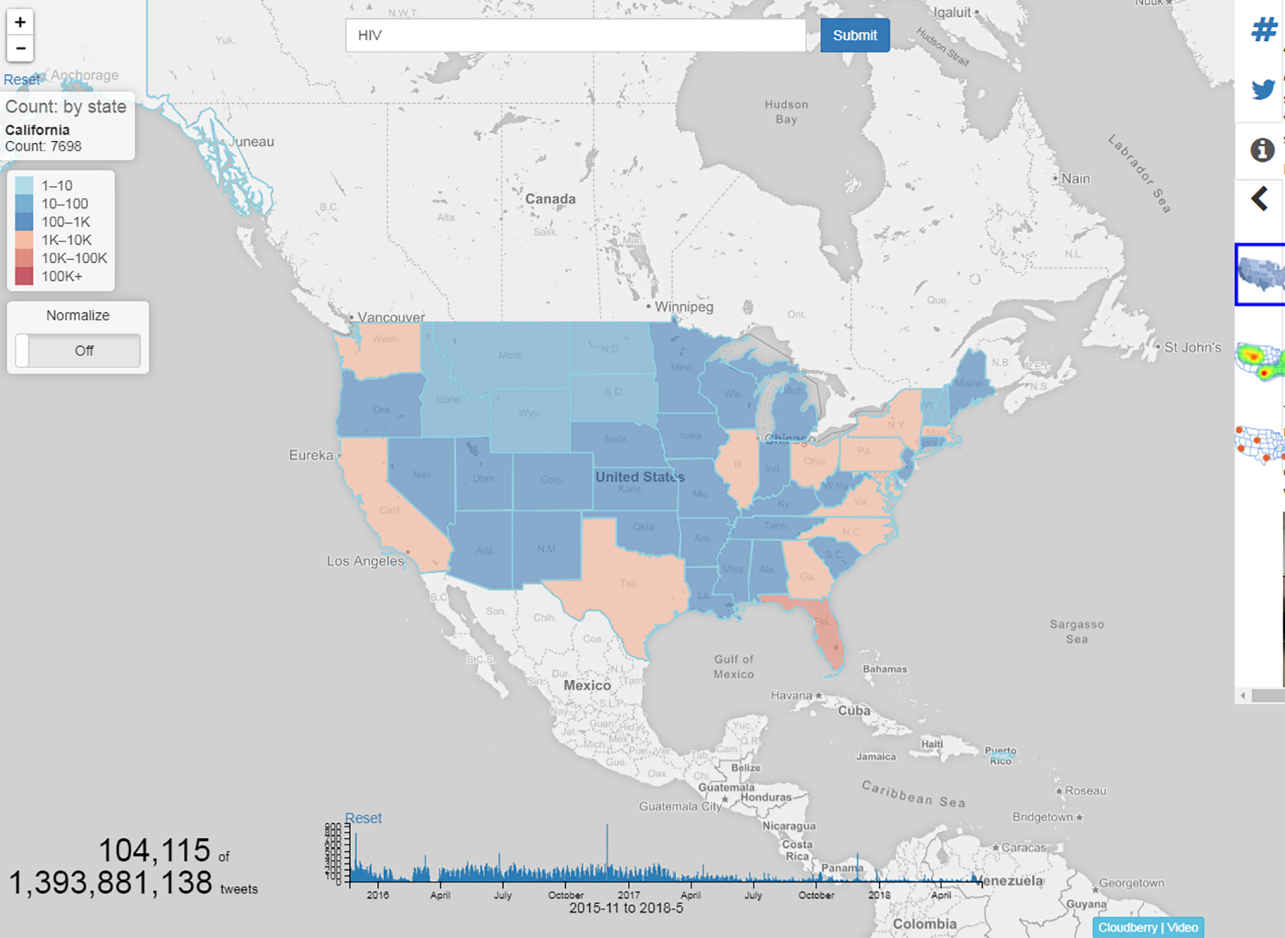
Map of social media using the keyword “HIV” in the United States. Image from UCIPT’s HIV ChatterMap tool.

Although the data in this study were relatively small compared to other “big data” studies, such as genomics studies, we refer to it this way because Google Trends data are supported by an average of 3.5 billion queries per day [25] submitted by large numbers of Web users over time. In addition we are modelling multidimensional data (from Google Trends and state HIV data), which is a key characteristic of “big data” research. Our goal is for these types of methodologies to eventually be able to be applied on Google Trends data in real-time in an effort to provide public health organizations with a way to monitor health crises on a day to day basis, such as through visualizations tracking the changing trends in potential new HIV cases diagnoses. Fig 2 represents an example of implications of how these types of models are currently being used by our team to provide maps for public health organizations and researchers. Visualizing social data with keywords of interest (e.g. HIV-related keywords) can help public health organizations track and predict the spread of diseases and plan more effective interventions. We are currently working with public health departments based on the research and models in this study to provide these types of visualization maps and tools to them so they can learn about people’s HIV-related discussions and predict new case diagnoses.

This study has limitations. First, we are limited by data, including a limited number of HIV cases and number of years that data were publicly reported by AIDSVu. Second, we are unable to define a “Google Trend” other than relative to other Google search terms. Google does not typically provide data on the number of searches, but rather provides researchers with data on the relative volume of searches [20]. Third, the keywords were taken from previous research using Twitter keywords. As people use Google and Twitter for different purposes they might use different keywords to talk about HIV-related topics on Twitter than for Google. Instead of using the same set of keywords across different technology data sites, future research can involve interviewing participants on the specific words they use on social media in order to create a model that is more specific to the type of technology used. Fourth, it is unknown whether searches for HIV-related behaviors and/or symptoms are associated with actual HIV infection within that individual. For example, individuals might be searching about sexual risk behaviors, but not engaging in those behaviors in a way that would actually put them at risk. Similarly, individuals might be searching for (non-clinically correct) signs and symptoms of HIV, reducing the likelihood that their search was linked to their HIV status. While this is an interesting topic for future research, we believe this is minor limitation to this study, as we found a pattern that associates searching for HIV-risk behaviors and HIV outcomes at the population, or epidemiologic level, rather than individual level. This information is therefore still actionable at the broader population level even if not at the individual level. Finally, Google Trends data are not available at the city level, limiting the ability for more targeted analyses.

## Conclusion

This study suggests that internet search data could be used as an additional tool for HIV surveillance and prediction. Methods of using Google search data and social media data for public health surveillance are increasingly being incorporated into public health efforts. These approaches are not meant to replace traditional public health surveillance systems, but may provide an additional tool that can be used to combat the spread of diseases, such as HIV.

## Supporting information

S1 Table The number of HIV cases and the predicted number for each state in 2011–2014. (DOCX)
