# Supplementary material for: Using search engine big data for predicting new HIV diagnoses
Source: PLoS One. 2018 Jul 12;13(7):e0199527. doi: 10.1371/journal.pone.0199527 (PMC6042696; doi:10.1371/journal.pone.0199527)
Supplement: S1 Table — (DOCX) [file pone.0199527.s001.docx]

|  | **2011** | | **2012** | | **2013** | | **2014** | |  |
| --- | --- | --- | --- | --- | --- | --- | --- | --- | --- |
| **State** | **Number of HIV cases** | **Prediction** | **Number of HIV cases** | **Prediction** | **Number of HIV cases** | **Prediction** | **Number of HIV cases** | **Prediction** | **RMSE** |
| **AK** | 24 | 40.69 | 29 | 25 | 24 | 30.28 | 41 | 24.87 | 12.19 |
| **AL** | 708 | 680.81 | 684 | 695.34 | 664 | 679.16 | 699 | 663.3 | 24.36 |
| **AR** | 246 | 239.4 | 254 | 248 | 270 | 256.16 | 340 | 272.49 | 34.75 |
| **AZ** | 574 | 605.63 | 644 | 552.64 | 728 | 627.67 | 798 | 719.44 | 79.99 |
| **CA** | 5226 | 5114.96 | 5280 | 4854.55 | 4958 | 5058.27 | 5533 | 4715.46 | 466.85 |
| **CO** | 380 | 415.72 | 386 | 349.45 | 326 | 382.99 | 401 | 319.99 | 55.74 |
| **CT** | 359 | 413.26 | 299 | 357.18 | 335 | 298.4 | 305 | 340.39 | 47.23 |
| **DE** | 116 | 122.51 | 139 | 114.93 | 119 | 142.61 | 132 | 121.72 | 17.93 |
| **FL** | 4777 | 4200.91 | 4664 | 4392.09 | 4662 | 4445.42 | 5332 | 4445.17 | 556.61 |
| **GA** | 2808 | 2586.68 | 2836 | 2590.86 | 2622 | 2744.84 | 2247 | 2545.91 | 231.04 |
| **HI** | 86 | 111.19 | 87 | 86.13 | 98 | 89.49 | 110 | 100.88 | 14.07 |
| **IA** | 119 | 114.55 | 119 | 116.68 | 126 | 121.18 | 104 | 128.89 | 12.92 |
| **ID** | 37 | 48.45 | 38 | 38.18 | 26 | 39.43 | 27 | 27.25 | 8.83 |
| **IL** | 1739 | 1656.57 | 1800 | 1601.29 | 1779 | 1750.81 | 1728 | 1736.09 | 108.57 |
| **IN** | 483 | 486.08 | 511 | 464.85 | 499 | 509.95 | 490 | 495.87 | 23.95 |
| **KS** | 140 | 144.09 | 156 | 141.48 | 153 | 156.95 | 138 | 155.77 | 11.82 |
| **KY** | 318 | 344.34 | 370 | 312.98 | 374 | 371.37 | 365 | 377.83 | 32.09 |
| **LA** | 1235 | 1087.35 | 1059 | 1242.19 | 1235 | 1041.57 | 1408 | 1241.54 | 173.56 |
| **MA** | 768 | 707.67 | 800 | 739.33 | 781 | 791.13 | 735 | 773.63 | 47.22 |
| **MD** | 1611 | 1721.97 | 1531 | 1448.62 | 1528 | 1457.05 | 1388 | 1484.08 | 91.34 |
| **ME** | 50 | 60.5 | 46 | 51.29 | 32 | 47.29 | 60 | 33.92 | 16.23 |
| **MI** | 797 | 762.46 | 820 | 772.54 | 793 | 810.91 | 837 | 783.79 | 40.62 |
| **MN** | 305 | 343.9 | 321 | 291.21 | 315 | 319.1 | 318 | 312.88 | 24.72 |
| **MO** | 536 | 560.31 | 548 | 516.12 | 483 | 546.44 | 500 | 482.14 | 38.57 |
| **MS** | 541 | 439.61 | 459 | 532.43 | 502 | 460.37 | 519 | 506.24 | 66.28 |
| **MT** | 21 | 21.22 | 20 | 20.87 | 23 | 21.06 | 16 | 24.53 | 4.4 |
| **NC** | 1494 | 1351.04 | 1293 | 1431.46 | 1362 | 1238.76 | 1416 | 1332.66 | 124.25 |
| **ND** | 12 | 15.14 | 11 | 12.9 | 20 | 11.61 | 24 | 21.37 | 4.77 |
| **NE** | 81 | 118.43 | 85 | 83.39 | 84 | 86.94 | 93 | 85.88 | 19.13 |
| **NH** | 41 | 53.11 | 48 | 41.09 | 34 | 47.77 | 41 | 34.99 | 10.25 |
| **NJ** | 1291 | 1372.43 | 1447 | 1225.1 | 1475 | 1411.73 | 1536 | 1443.26 | 130.84 |
| **NM** | 140 | 144.44 | 122 | 136.94 | 147 | 122.72 | 141 | 150.23 | 15.15 |
| **NV** | 388 | 378.06 | 375 | 377.25 | 457 | 373.78 | 463 | 455.79 | 42.08 |
| **NY** | 4009 | 3751.33 | 3797 | 3677.73 | 3558 | 3640.48 | 3817 | 3449.27 | 235.94 |
| **OH** | 1062 | 912.58 | 1044 | 1025.31 | 1074 | 1026.24 | 1005 | 1054.38 | 82.77 |
| **OK** | 321 | 288.71 | 294 | 314.14 | 347 | 294.84 | 334 | 348.89 | 33.14 |
| **OR** | 244 | 230.32 | 271 | 243.4 | 230 | 268.11 | 244 | 230.94 | 25.36 |
| **PA** | 1425 | 1386.68 | 1473 | 1313.67 | 1409 | 1438.21 | 1322 | 1377.69 | 87.77 |
| **RI** | 102 | 110.73 | 77 | 103.35 | 88 | 76.5 | 101 | 92.28 | 15.65 |
| **SC** | 761 | 783.58 | 723 | 749.41 | 748 | 712.64 | 841 | 741.85 | 55.43 |
| **SD** | 22 | 35.74 | 25 | 21.95 | 34 | 25.97 | 28 | 35.65 | 8.96 |
| **TN** | 862 | 782.3 | 882 | 832.5 | 811 | 870.73 | 808 | 804.94 | 55.64 |
| **TX** | 4404 | 4514.52 | 4496 | 4260.13 | 4547 | 4514.36 | 4817 | 4520.36 | 198.07 |
| **UT** | 104 | 76.89 | 115 | 101.75 | 110 | 116.79 | 120 | 112.26 | 15.95 |
| **VA** | 958 | 997.46 | 967 | 917.41 | 1022 | 936.79 | 983 | 994.05 | 53.39 |
| **VT** | 12 | 24.59 | 14 | 11.76 | 15 | 14.73 | 19 | 16.17 | 6.56 |
| **WA** | 496 | 536.71 | 509 | 470.13 | 468 | 504.88 | 455 | 464.37 | 33.98 |
| **WI** | 250 | 255.9 | 223 | 237.04 | 255 | 226 | 230 | 257.43 | 21.37 |
| **WV** | 93 | 76.79 | 84 | 95.17 | 78 | 86.97 | 96 | 81.47 | 13.04 |
| **WY** | 15 | 19.07 | 7 | 15.31 | 16 | 7.29 | 12 | 16.85 | 6.8 |
